# From coal biodesulfurization to mine-soil remediation: a critical review of microbial sulfur transformation in coal-impacted environments

**DOI:** 10.3389/fbioe.2026.1888081

**Published:** 2026-07-23

**Authors:** Nuraly Akimbekov, Kuanysh Tastambek, Ilya Digel, Alan Aimagambetov, Marzhan Kozhakhmetova, Nazym Altynbay, Dinara Sherelkhan

**Affiliations:** 1 Sustainability of Ecology and Bioresources, Al-Farabi Kazakh National University, Almaty, Kazakhstan; 2 Ecology Research Institute, Khoja Akhmet Yassawi International Kazakh-Turkish University, Turkistan, Kazakhstan; 3 Institute for Bioengineering (IFB) at FH Aachen University of Applied Sciences, Julich, Germany

**Keywords:** acid mine drainage, coal biodesulfurization, coal gangue, mine soil, pyrite, Soil remediation, sulfate-reducing bacteria, sulfur oxidation

## Abstract

Coal biodesulfurization has traditionally been regarded as a pre-combustion clean-coal technology; however, its connections to sulfur cycling and remediation in coal-impacted soils have not been sufficiently integrated. This review addresses this gap by examining microbial sulfur transformations in coal, coal gangue, mine spoil, acid mine drainage systems, and reclaimed soils. The discussion encompasses pyritic sulfur biooxidation, organic sulfur biotransformation, sulfate reduction and metal sulfide precipitation, rhizosphere-mediated sulfur cycling, and the reuse of coal mine solid wastes in reclamation contexts. Drawing on these lines of evidence, the review proposes a systems-level, risk-controlled sulfur management framework that conceptualizes coal treatment and mine-soil remediation as interconnected microbial, mineral, hydrological, and ecological processes. The synthesis demonstrates that sulfur-transforming microorganisms may facilitate sulfur removal, waste stabilization, and vegetation recovery, or alternatively promote acidification, sulfate export, and metal mobilization. These outcomes depend on factors such as matrix accessibility, oxygen exposure, redox stability, hydrologic connectivity, buffering capacity, sulfur loading, and labile carbon availability. Future research should progress beyond isolated microbial screening and instead prioritize matrix-aware, field-validated, and risk-based strategies that integrate sulfur mass balance, contaminant mobility, and long-term reclamation performance.

## Graphical Abstract

**Figure:**
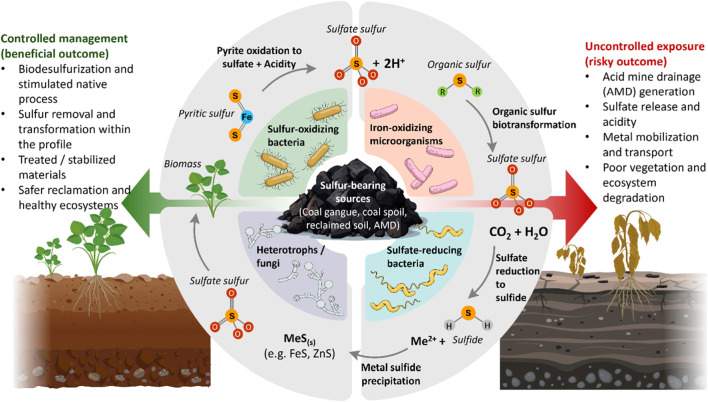
From coal sulfur to soil outcome.

## 1 Introduction

Coal remains a significant energy and industrial resource in many regions; however, its sulfur content poses substantial environmental and operational challenges. During combustion, sulfur-containing compounds in coal are converted into sulfur oxides, contributing to air pollution and acid deposition. Even before combustion, sulfur-bearing minerals and organic sulfur groups can affect coal storage stability, increase the risk of spontaneous combustion, and complicate mineral processing and downstream waste management. Consequently, coal desulfurization using physical, chemical, and biological methods has been extensively investigated (Sekhohola-Dlamini et al., 2025; Çelik et al., 2019). A recent comprehensive review by Dinh et al. indicates that research continues to evaluate these pretreatment strategies and that integrative approaches may improve sulfur removal efficiency while reducing operational costs and wastewater production (Dinh et al., 2025).

Coal biodesulfurization is frequently characterized as a cleaner alternative to chemical treatments, because microorganisms can oxidize or transform sulfur under relatively mild conditions. Previous reviews on microbial desulfurization have mainly focused on sulfur-removing microorganisms and their associations with specific inorganic and organic sulfur fractions (Koyunoğlu and Karaca, 2023; Kotelnikov et al., 2020). However, this perspective is somewhat restrictive. In actual mining environments, coal sulfur represents not only a fuel-quality concern but also a driver of significant soil and ecosystem challenges. Sulfur-rich coal residues, coal gangue, mine spoil, and acid mine drainage affect soil acidity, redox chemistry, sulfate accumulation, plant nutrition, metal mobility, microbial succession, and land reclamation outcomes (Yu et al., 2025; Ma et al., 2020; Zhang et al., 2025).

Importantly, the present review is not intended to duplicate these earlier syntheses (Sekhohola-Dlamini et al., 2025; Çelik et al., 2019; Dinh et al., 2025; Koyunoğlu and Karaca, 2023; Kotelnikov et al., 2020). Its unique contribution is fourfold. *First,* it shifts the focus from coal as an isolated feedstock to coal-impacted environments that include coal gangue, mine spoil, Technosols, drainage waters, and rhizosphere soils. *Second,* it integrates oxidative sulfur removal with reductive sulfur stabilization, thereby linking coal biodesulfurization to mine-soil remediation and reclamation. *Third,* it emphasizes matrix accessibility, sulfur mass balance, hydrological transport, and field scalability as critical but underrepresented controls on microbial performance. *Fourth,* it advances a coupled soil-mine biogeochemical framework that connects reactor-scale desulfurization, waste conditioning, and ecosystem recovery. In this way, the review moves beyond a fuel-centered inventory of microorganisms and pathways toward a systems-level, matrix-aware, and remediation-oriented synthesis.

This review therefore frames coal biodesulfurization as a process that should be considered alongside soil science. Microbial processes that are beneficial for coal sulfur removal may become detrimental when they proceed uncontrolled in mine soils. While pyrite oxidation facilitates sulfur removal from coal, it can also generate acidity and mobilize metals in mine spoil (Nancucheo and Johnson, 2011; Monterroso and Macías, 1998). Sulfur-oxidizing microorganisms can enhance sulfate availability for plants; however, excessive oxidation of sulfide minerals may destabilize reclaimed soils (Nguyen et al., 2022; Koźmińska et al., 2024). Sulfate-reducing microorganisms have the potential to immobilize metals and neutralize acidity, yet their efficacy depends on organic carbon availability, anaerobic microsites, plant root activity, and soil structure (Ayangbenro et al., 2018; Yang et al., 2023). Adopting a soil-science perspective may help transform coal biodesulfurization from a laboratory-based fuel-cleaning technique into a field-relevant, comprehensive and environmentally integrated management strategy.

To address the knowledge gap identified above, the remainder of this review is organized as follows. Section 2 establishes the conceptual framework of coal sulfur as a coupled soil-mine biogeochemical challenge. Section 3 defines the major sulfur forms and transformations in coal and coal-impacted environments, providing the chemical basis for subsequent discussion. Section 4 reviews the principal microbial platforms for pyritic and organic sulfur removal, whereas Section 5 extends these processes to coal-impacted soil systems by examining acid mine drainage, sulfate reduction, plant sulfur nutrition, and rhizosphere recovery. Section 6 identifies coal gangue as a practical interface between biodesulfurization and soil remediation, Section 7 discusses methodological priorities such as sulfur mass balance and field relevance, and Sections 8–10 synthesize the major research gaps, future directions, and broader implications. Together, these sections address the identified knowledge gap by moving from isolated microbial mechanisms toward an integrated framework for controlled sulfur management across coal, soil, water, and reclamation systems.

After this brief positioning relative to earlier reviews, the sections that follow prioritize evidence from primary reactor, field, and remediation studies wherever possible; review articles are used mainly to define the broader literature landscape, not as substitutes for original experimental or observational findings.

## 2 Conceptual framework: coal sulfur as a soil-mine biogeochemical challenge

The central concept of this review is that coal sulfur should be understood as part of an interconnected biogeochemical transformation cascade: coal sulfur → microbial transformation → sulfate production, acidity generation, iron cycling → soil chemical change → metal mobility → plant response → reclamation outcome. This framework is synthesized from recurring patterns reported in three complementary bodies of primary evidence: controlled biodesulfurization studies showing that pH, particle size, sulfur form, and coal-matrix accessibility control sulfur removal (Ai et al., 2026; He et al., 2012; Xu et al., 2022); field and site studies showing that exposure of coal gangue and mine spoil to oxygen and water drives sulfate release, acidity generation, and shifts in soil and vegetation (Ma et al., 2020; Monterroso and Macías, 1998; Zhang et al., 2025; Dong et al., 2023); and remediation experiments showing that sulfate reduction, metal sulfide precipitation, and conditioned gangue amendments can stabilize contaminants and improve plant performance (Wang X. et al., 2021; Dong et al., 2024; Li S. et al., 2024). Accordingly, the cascade is used here as a simplified organizing scaffold for linked processes across scales rather than as a claim that every step proceeds sequentially and identically at every site. In real systems, feedback loops remain important: plant roots alter pH and redox microsites, iron minerals adsorb or release sulfate and metals, and microbial consortia respond dynamically to shifts in both pH and carbon availability.

Coal biodesulfurization research typically aims to remove sulfur from solid coal. In contrast, soil remediation focuses on managing sulfur to control acidity, salinity, and toxic metal mobility, while enhancing soil fertility and plant establishment. This distinction creates a complex but valuable intersection between the two disciplines.

For example, primary studies show that coal gangue can behave both as a source of sulfur-related risk and as a conditional reclamation material. Field investigations have shown that abandoned or weathered gangue can enrich surrounding soils in sulfur and iron, alter vegetation, and release heavy metals during oxidation (Dong et al., 2026). In contrast, application-oriented experiments indicate that, after appropriate conditioning or biological treatment, coal gangue can improve plant establishment and selected soil properties in reclaimed substrates or saline-alkali soils (Gao et al., 2024; Sun Y. et al., 2025). These contrasting observations provide the empirical basis for integrating coal biodesulfurization and soil-science perspectives.

A soil-science framework shifts attention from single-strain microbial desulfurization performance to the broader, matrix-dependent environmental context. Factors such as soil pH, redox potential, moisture, texture, organic matter, root exudates, iron minerals, sulfate concentration, and microbial consortia collectively influence sulfur cycling (Abdul Rahman et al., 2021; Patsis et al., 2025). Consequently, field-scale sulfur transformation cannot be inferred directly from laboratory desulfurization efficiency alone; it also requires assessment of matrix composition, buffering capacity, hydrology, redox heterogeneity, contaminant release, and plant-microbe feedbacks.

Across the studies reviewed here, whether sulfur-transforming microorganisms act beneficially or detrimentally is controlled less by microbial identity alone than by the surrounding physicochemical setting. Five variables are especially important: (1) oxygen availability and redox stability, which determine whether oxidation or reduction dominates; (2) water saturation and hydrologic connectivity, which regulate sulfate export and contaminant transport; (3) pH buffering capacity and alkalinity, which determine whether acidity is neutralized or amplified; (4) sulfur loading and substrate accessibility, especially pyrite exposure and coal-matrix structure; and (5) labile organic carbon availability, which governs the feasibility of sulfate reduction and metal sulfide stabilization (Moyo et al., 2019; Tran et al., 2021; Li et al., 2024a; Wang Z. et al., 2021; Novak et al., 2018). Beneficial outcomes are most likely when these variables are managed so that sulfur oxidation is spatially confined, sulfate and metals are captured or reused, and reductive stabilization occurs in persistently anoxic, carbon-sufficient microsites. Detrimental outcomes are more likely when pyrite-rich materials remain exposed to repeated wetting and aeration, buffering capacity is low, and oxidation products are freely exported to surrounding soils and waters (Li et al., 2024a; Novak et al., 2018; Li et al., 2023; Jiao et al., 2023).

## 3 Sulfur forms and transformations in coal and coal-impacted soils

Sulfur in coal is commonly classified into inorganic and organic fractions. The inorganic fraction is primarily associated with pyrite and sulfate minerals, such as gypsum and jarosite, whereas organic sulfur occurs as thiophenic, sulfidic, sulfoxide, sulfone, and thiol-type structures within the coal matrix (Table 1) (Ai et al., 2026; Moyo et al., 2019; Calkins, 1994).

**TABLE 1:** Sulfur forms and microbial relevance in coal-impacted environments.

Sulfur form	Main location	Important microbial process	Environmental meaning	Ref
Pyritic sulfur	Coal, coal gangue, mine spoil	Oxidation by acidophilic Fe/S oxidizers	Useful for biodesulfurization, but risky for acid generation	Li et al. (2023), Jiao et al. (2023)
Sulfate sulfur	Oxidized coal residues, soil solution, AMD	Plant uptake or sulfate reduction	Can support nutrition or contribute to salinity/acidity	Wang Y.-W. et al. (2023), Prayuenyong (2002), Malviya et al. (2022)
Thiophenic sulfur	Organic coal matrix	4 S-pathway-type biotransformation	Important for organic sulfur removal, difficult in real coal	Martínez et al. (2022)
Sulfides/sulfoxides/sulfones	Coal organic matter	Oxidation, enzymatic transformation	Depends strongly on coal matrix accessibility	Ai et al. (2026)
Sulfate reduced to sulfide	Anaerobic soils, wetlands, treatment systems	Sulfate-reducing bacteria	Can immobilize metals as sulfides	Yang et al. (2023), Dong et al. (2024)

In coal-impacted soils, sulfur species are more dynamically transformed because they are exposed to fluctuating redox conditions, water flow, plant uptake, and microbial activity. Pyrite and other sulfide minerals oxidize upon exposure to oxygen and water, generating sulfate, acidity, and coupled Fe^2+^/Fe^3+^ redox cycling (Wang X. et al., 2021). Sulfate may be leached, adsorbed, assimilated by plants, reduced by sulfate-reducing bacteria, or incorporated into organic sulfur pools (Tran et al., 2021). In regions with high-sulfur coal mining, sulfur is also associated with coal gangue, mine spoil, and secondary minerals (Dong et al., 2023).

**FIGURE 1:**
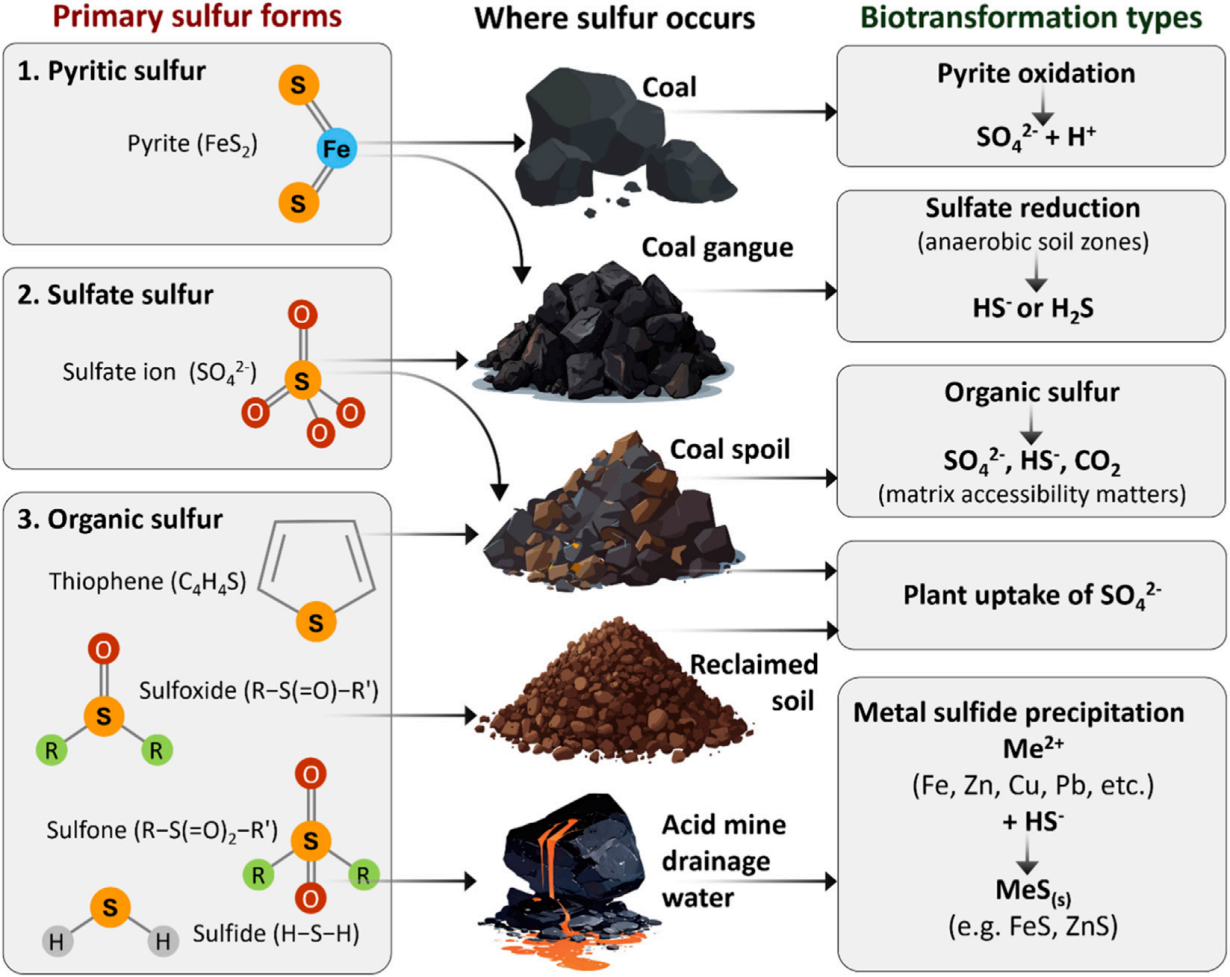
Sulfur forms in coal and coal-impacted environments. Major sulfur pools in coal-impacted systems include pyritic sulfur (FeS_2_), sulfate sulfur (SO_4_^2-^), and organic sulfur species such as thiophenic sulfur, sulfides, sulfoxides, and sulfones. These forms occur across coal, coal gangue, mine spoil, reclaimed soils, and drainage waters, and their fate depends on oxidation-reduction conditions, matrix accessibility, hydrological transport, and microbial activity. Key transformations include pyrite oxidation, organic sulfur biotransformation, sulfate transport and plant uptake, sulfate reduction to sulfide, and metal sulfide precipitation.

The major sulfur forms and their dominant transformations across coal, coal gangue, mine spoil, reclaimed soil, and drainage waters are summarized in Figure 1 **.**

Wang et al. illustrate the complexity of sulfur distribution in high-sulfur coal mining areas in China. They show that sulfur is widely present in coal gangue, frequently complexed with metals as sulfide minerals, and that pyrite is a primary sulfur source in coal gangue. The study further indicates that mining activities disturb sulfur equilibrium, increase sulfur reactivity, and alter sulfur-driven biogeochemical cycles (Wang Y.-W. et al., 2023). These findings support the view that sulfur in coal mine environments is not a static contaminant pool but a reactive component of coupled mineral, microbial, hydrological, and ecological processes.

## 4 Microbial platforms for coal biodesulfurization

### 4.1 Pyritic sulfur biooxidation

The most extensively studied route for coal biodesulfurization involves microbial oxidation of pyritic sulfur. Acidophilic iron- and sulfur-oxidizing bacteria, including *Acidithiobacillus ferrooxidans, Acidithiobacillus thiooxidans, Acidithiobacillus caldus,* and related species, facilitate the oxidation of Fe^2+^ and reduced sulfur species, such as elemental sulfur, thiosulfate, and sulfide minerals. In controlled coal treatment systems, this process effectively removes inorganic sulfur, particularly pyrite. However, in mine soils and coal gangue dumps, the same microbial activity can result in the formation of acid mine drainage (He et al., 2012; Wang et al., 2018; Singh et al., 2021).

Zhao et al. examined microbial sulfur removal from coal using *Acidithiobacillus ferrooxidans* under controlled laboratory conditions. Temperature, pH, particle size, and inoculum volume significantly influenced performance, and treatment reduced pyrite content, altered coal surface morphology, and increased the spontaneous-combustion temperature of the treated coal (Zhao et al., 2023). Quantitatively, under the reported optimum conditions (30 °C, 120 mesh, initial pH 2.0, and 15 mL inoculum), the maximum desulfurization rate reached 75.12%, whereas sulfur removal across the 16 orthogonal trials ranged from 32.65% to 68.22%. The same study also reported a 50 °C increase in coal ignition point and an increase in activation energy from 8,934.3 to 31,464.4 J mol^−1^ after treatment (Zhao et al., 2023).

From a soil-science perspective, the primary question is whether pyrite biooxidation is spatially and chemically controlled. It is beneficial when conducted in managed biodesulfurization reactors, where sulfate- and iron-rich effluents can be collected and treated (Ghassa et al., 2022). In contrast, uncontrolled biooxidation in coal gangue piles, mine spoils, or reclaimed soils can be detrimental (Li et al., 2024a). Thus, microbial pyrite oxidation is both a tool for sulfur removal and a potential driver of soil acidification. In other words, pyrite biooxidation is beneficial mainly under *ex situ* or reactor-based conditions with leachate capture, residence-time control, and post-treatment neutralization, but becomes detrimental in exposed gangue or spoil where oxygen diffusion, rainfall infiltration, and low neutralization capacity allow acidity and dissolved metals to accumulate and migrate (Li et al., 2024a; Wang Y. et al., 2021; Novak et al., 2018; Ghassa et al., 2022).

### 4.2 Organic sulfur biotransformation and the 4S pathway

Organic sulfur is more challenging to remove than pyritic sulfur due to its chemical integration within the coal matrix. Dibenzothiophene (DBT) is frequently employed as a model compound for thiophenic sulfur in numerous studies. The predominant microbial pathway for DBT biodesulfurization is the 4 S pathway, which converts DBT to 2-hydroxybiphenyl via sulfur-specific oxidation and cleavage reactions catalyzed by enzymes such as DszC, DszA, DszB, and DszD (Xu et al., 2022; Martínez et al., 2022).

The 4S pathway is significant because it removes sulfur while preserving the integrity of the hydrocarbon skeleton. This characteristic is advantageous in fuel biodesulfurization, as it maintains the fuel’s value (Mishra et al., 2016). However, applying findings from DBT-based studies to actual coal biodesulfurization remains challenging (Das et al., 2024). Real coal possesses a heterogeneous, cross-linked organic matrix in which sulfur groups may be sterically protected or physically inaccessible to microbial enzymes. Recent work illustrates this constraint. A study by C. Ai et al., utilizing *Pseudomonas putida,* demonstrated that two coal samples with distinct matrix structures exhibited markedly different organic sulfur removal rates: 60.9% for one sample and 17.6% for the other. The authors attributed these differences to variations in coal aromaticity, condensation degree, steric hindrance, and enzyme-substrate compatibility (Ai et al., 2026). This finding is highly relevant to the present review. It indicates that future biodesulfurization research should address not only the question of which microorganism removes sulfur, but also which microorganism removes specific sulfur forms from particular coal matrices under defined soil or reactor conditions. Expressed differently, the more accessible coal exhibited a 43.3 percentage-point advantage and approximately 3.5-fold higher organic sulfur removal than the less accessible matrix, despite treatment with the same microbial species (Ai et al., 2026).

### 4.3 Heterotrophic fungal and bacterial consortia

While acidophilic bacteria are predominant in pyritic sulfur research, fungi and heterotrophic bacteria are also significant, particularly regarding low-rank coal, organic sulfur, and coal biodegradation. Fungi produce extracellular enzymes, organic acids, biosurfactants, and oxidative metabolites that can modify coal surfaces or solubilize coal-derived organic matter (Nussipov et al., 2026; Akimbekov et al., 2022; Nin et al., 2025). Heterotrophic bacteria transform organic sulfur compounds or function within consortia, where one organism alters the coal matrix and another oxidizes or assimilates sulfur (Chen et al., 2025; Li et al., 2024b).

Recent primary studies likewise support a broader role for consortium-based systems. Indigenous and exotic microbial communities can desulfurize high-sulfur coal and alter its physicochemical properties (Ye et al., 2018), sulfur-conversion functional communities from biogas liquid can participate in coal degradation (Li et al., 2024b), and biologically treated gangue or coal gangue–microalgae matrices can improve plant establishment in reclaimed substrates (Ye et al., 2018; Wang et al., 2024). Together, these findings indicate that consortium-driven coal transformation can be linked directly to soil rehabilitation rather than being viewed only through the lens of isolated coal-cleaning performance.

In this context, fungal and heterotrophic systems should be considered not only as coal-cleaning agents but also as soil-forming mediators. Their capacity to generate humic-like substances, enhance organic matter cycling, and interact with plant roots is potentially more valuable for mine-soil remediation than for rapid industrial desulfurization.

**FIGURE 2:**
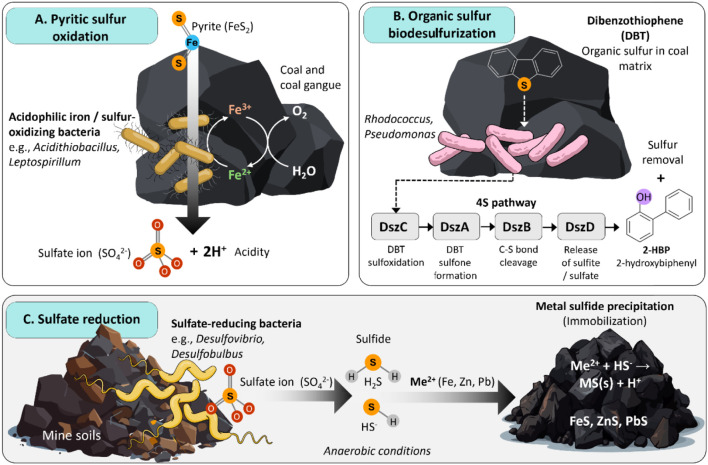
Microbial pathways relevant to this review. Conceptual summary of three major sulfur-transforming pathways in coal-impacted environments: pyritic sulfur oxidation **(A)**, organic sulfur biodesulfurization **(B)**, and sulfate reduction to sulfide with metal sulfide precipitation **(C)**. These pathways link coal biodesulfurization with mine-soil sulfur cycling and remediation.

The principal microbial pathways that connect coal biodesulfurization with sulfur cycling and stabilization in mine soils are summarized in Figure 2 **.**

## 5 Microbial sulfur transformations in coal-impacted soil systems

### 5.1 Sulfur oxidation and acid mine drainage

Acid mine drainage exemplifies uncontrolled geochemical–microbial sulfur transformation. Field observations and site studies from coal mine environments consistently show that exposing pyrite-bearing materials to oxygen and water generates acidic, sulfate-rich, metal-laden drainage (Zhang et al., 2025; Li et al., 2023; Wang Y. et al., 2021).

This mechanism establishes a direct link between coal biodesulfurization and soil science. In a coal biodesulfurization reactor, microbial pyrite oxidation is advantageous as it facilitates sulfur removal from coal (Dinh et al., 2025; Yuan et al., 2025). By contrast, when the same reaction proceeds in an unmanaged mine spoil pile, it can decrease soil pH, increase sulfate concentrations, and mobilize iron, manganese, aluminum, and potentially toxic trace elements (Novak et al., 2018; Garbacz et al., 2025). The distinction lies not in the microbial pathway, but in the environmental context and the degree of control over reaction rates, products, transport, and containment.

**FIGURE 3:**
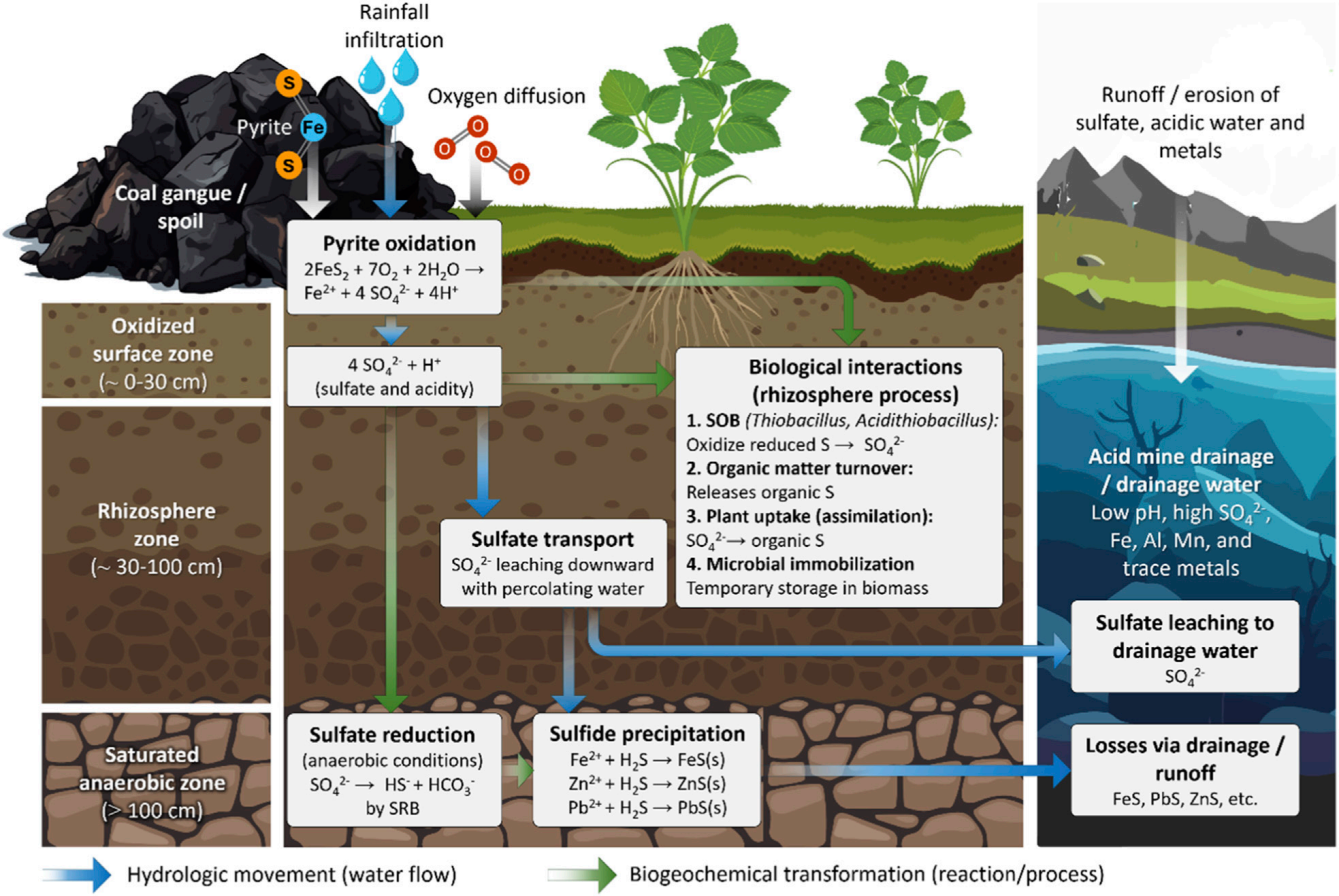
Environmental sulfur cycle in coal-impacted soils. Conceptual model of sulfur cycling across oxidized surface horizons, rhizosphere zones, saturated anaerobic layers, and drainage pathways in coal-impacted landscapes. Pyrite oxidation in coal, coal gangue, or mine spoil produces sulfate and acidity; sulfate is then transported by infiltrating water, adsorbed or desorbed from soil particles, assimilated by plants and microorganisms, mineralized from organic matter, or reduced to sulfide in anaerobic microsites by sulfate-reducing bacteria. The resulting sulfide may precipitate dissolved metals as secondary metal sulfides or be reoxidized under oxic conditions, while sulfate and acidity may also be exported to mine drainage systems. Sulfur fate therefore depends on redox gradients, hydrology, microbial activity, and plant–soil interactions.

Therefore, mine-soil remediation strategies should not focus solely on maximizing sulfur oxidation. Instead, remediation should regulate sulfur transformation to achieve a balance among sulfur removal, acidity neutralization, and metal immobilization. A conceptual overview of sulfur turnover across oxidized, rhizosphere, and anaerobic zones in coal-impacted soils is presented in Figure 3 **.**

### 5.2 Sulfate-reducing bacteria and metal immobilization

Sulfate-reducing bacteria (SRB) provide a key counter-process to sulfur oxidation. Under anaerobic conditions and in the presence of appropriate electron donors, SRB reduce sulfate to sulfide (Bagheri Novair et al., 2024; Xie et al., 2025). The resulting sulfide can precipitate metals as relatively insoluble metal sulfides, and sulfate reduction can also enhance alkalinity. In coal mine soils, SRB therefore contribute to metal immobilization and acidity regulation (Yang et al., 2023; Mafane et al., 2025).

A study by Yang et al. on coal mine-impacted soils reported that an SRB consortium dominated by *Desulfovibrio* and *Desulfobulbus* improved immobilization of metals such as Zn, Cu, As, and Pb, with metal residual forms increasing substantially. The study also emphasized that SRB interactions with plants and native soil microbiota remain complex and need further investigation (Yang et al., 2023). These effects were quantitatively substantial: SRB treatment reduced sulfate values by 52.1% (32.14 ± 2.86 to 15.38 ± 1.36), lowered ORP from 226.85 ± 14.64 to 67.42 ± 4.04 mV, and increased pH from 6.25 ± 0.53 to 7.12 ± 0.28. It also raised the combined residual fraction of key metals from 23.47% to 75.98%; the water-soluble fraction of Zn fell from 35.25% to 3.44%, while the residual fraction of Cu increased from 27.49% to 65.39% (Yang et al., 2023).

Coal biodesulfurization research often focuses on oxidation, but mine-soil remediation may require both oxidation and reduction. For this review, SRB are important because they broaden the discussion from a purely oxidative desulfurization perspective to a full sulfur-cycle framework. Oxidation can transform sulfide sulfur into sulfate; reduction can remove sulfate and immobilize metals. A practical remediation system may therefore require spatial or temporal separation of these processes.

Accordingly, SRB-mediated stabilization is most favorable in water-saturated, weakly acidic to circumneutral microsites where oxygen intrusion is limited, sulfate is available, and labile organic carbon or other electron donors are sufficient to sustain reduction (Ayangbenro et al., 2018; Yang et al., 2023; Bagheri Novair et al., 2024; Mafane et al., 2025). By contrast, the long-term benefit of sulfate reduction decreases where carbon is depleted, redox conditions oscillate, or newly formed sulfides are later re-oxidized during drainage or drying events (Tran et al., 2021; Bagheri Novair et al., 2024).

### 5.3 Sulfur-oxidizing bacteria and plant sulfur nutrition

Sulfur oxidation is not universally detrimental. In agricultural and reclaimed soils, this process can enhance sulfate availability, the main plant-available form of sulfur. Sulfur-oxidizing bacteria originating from coal mine environments may contribute to plant growth, provided that soil acidity and metal mobility are effectively managed (Kumar et al., 2024; Yi et al., 2021).

Malviya et al. investigated sulfur-oxidizing bacteria isolated from coal mines and identified strains from the genera *Stenotrophomonas*, *Rhizobium*, *Bacillus*, and *Paenibacillus*. Several isolates reduced pH in thiosulfate medium and generated sulfate. The study assessed their capacity to enhance sulfur uptake and promote growth in pigeonpea (Malviya et al., 2022). The quantitative magnitude of this response is notable. The best-performing isolate produced 311.43 mg L^−1^ sulfate after 21 days; in non-sterilized soil after 60 days, inoculation increased pigeonpea shoot length from 43.25 to 59.92 cm and shoot dry weight from 3.50 to 6.95 g relative to the untreated control (Malviya et al., 2022).

These findings support a strong connection between coal biodesulfurization and soil fertility. Microorganisms from coal mine environments may have dual relevance: facilitating coal desulfurization and enhancing sulfur nutrition in plants cultivated on reclaimed or sulfur-deficient soils (Yang et al., 2023). However, it is essential to distinguish this beneficial application from pyrite-induced acidification. While controlled sulfur oxidation that releases moderate sulfate levels can support plant growth, uncontrolled sulfide oxidation may result in soil degradation.

Conversely, plant-beneficial sulfur oxidation is most likely in alkaline or soda-saline soils with measurable neutralization capacity, moderate sulfur loading, active root uptake, and limited hydrologic export of sulfate and metals (Nguyen et al., 2022; Koźmińska et al., 2024; Malviya et al., 2022; Bao et al., 2016). In weakly buffered mine spoils or metal-rich gangue, the same oxidation processes can become detrimental because sulfate and acidity are produced faster than they can be assimilated, neutralized, or retained within the soil profile (Wang Y. et al., 2021; Novak et al., 2018; Garbacz et al., 2025).

### 5.4 Afforestation, rhizosphere effects, and sulfur-cycle recovery

Vegetation exerts a significant influence on sulfur cycling within coal-impacted soils. Plant roots modify soil oxygen levels, moisture content, organic carbon availability, microbial community composition, and the distribution of sulfur metabolites (Pan et al., 2026). In regions affected by high-sulfur coal mining, afforestation can alter soil chemistry, microbial diversity, metal distribution, electrical conductivity, and sulfur-metabolite profiles (Sun J. et al., 2025).

Wang et al. found that plant cover under different afforestation patterns in high-sulfur coal mining areas promoted sulfur-related microorganisms such as *Desulfovibrio, Acidiferrobacter, Acidithiobacillus,* and *Thiomonas*. They also reported increases in pH, organic carbon, total nitrogen, and available nitrogen, together with lower electrical conductivity in shallow soil layers (Wang Y.-W. et al., 2023).

This finding shows that mine-soil sulfur cycling is not only a geochemical process but also part of ecological succession. Reclamation success depends on how plants, microbes, minerals, and sulfur species co-develop over time.

**FIGURE 4:**
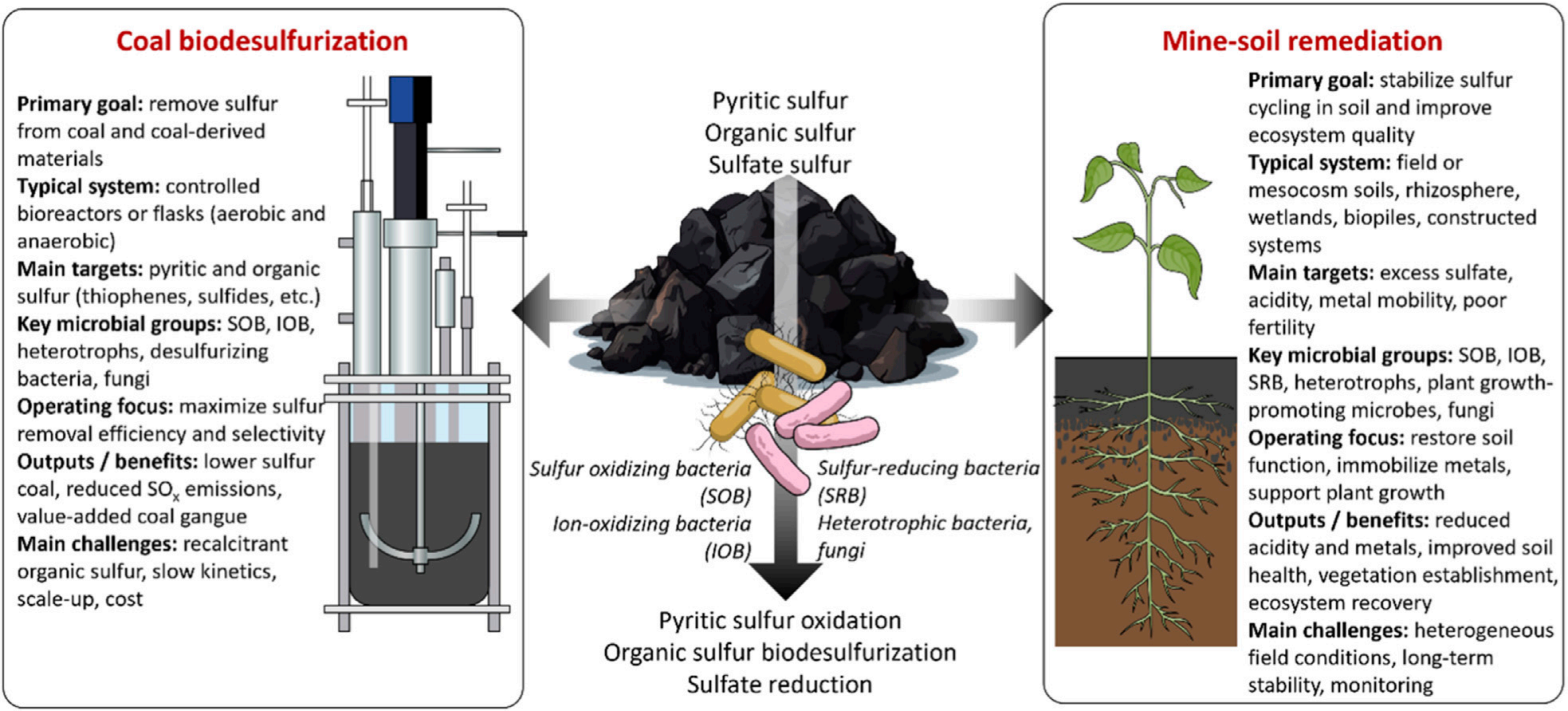
Coal biodesulfurization versus mine-soil remediation: same microbes, different goals. Side-by-side comparison showing that similar sulfur-transforming microbial groups operate in both engineered coal-treatment systems and coal-impacted soils, but are directed toward different outcomes: sulfur removal in the former and sulfur stabilization, metal control, and ecosystem recovery in the latter.

The contrast between coal biodesulfurization as an engineered sulfur-removal strategy and mine-soil remediation as a field-scale sulfur-management process is summarized in Figure 4 **.**

Taken together, the studies reviewed above indicate that “beneficial” versus “detrimental” sulfur transformation can be predicted from a limited set of environmental controls rather than from microbial identity alone. In practical terms, beneficial outcomes require managed redox zoning, adequate buffering, constrained sulfate export, and, for sulfate reduction, persistent anoxia with sufficient labile carbon; detrimental outcomes arise when sulfur-rich substrates are hydrologically connected, poorly buffered, intermittently oxidized, or allowed to re-oxidize after reductive stabilization. These contrasting conditions are summarized in Table 2.

**TABLE 2:** Environmental conditions that determine whether sulfur-transforming microorganisms produce beneficial or detrimental outcomes.

Process/Microbial guild	Beneficial conditions	Detrimental/Risk conditions	Likely outcome	Ref.
Pyrite oxidation by acidophilic Fe/S oxidizers	Contained or *ex situ* treatment; leachate capture; managed oxygen supply; post-treatment neutralization; limited off-site hydrologic connectivity	Exposed pyritic gangue/spoil; simultaneous oxygen diffusion and rainfall infiltration; low acid-neutralizing capacity; no effluent containment	Sulfur removal from coal vs. acid generation, sulfate release, and AMD formation	Li et al. (2024a), Wang Y. et al. (2021), Novak et al. (2018), Ghassa et al. (2022)
Sulfur oxidation for plant nutrition/pH adjustment	Moderately alkaline or saline soils; moderate sulfur loading; sufficient buffering; active plant uptake; managed drainage	Already acidic or weakly buffered soils; excessive sulfur loading; sulfate accumulation or export; vegetation stress	Improved sulfate nutrition and pH adjustment vs. acidification and metal mobilization	Nguyen et al. (2022), Koźmińska et al. (2024), Wang Y. et al. (2021), Novak et al. (2018), Malviya et al. (2022), Bao et al. (2016)
Sulfate reduction by SRB	Anaerobic or low-redox microsites; adequate sulfate; labile organic carbon/electron donors; limited oxygen intrusion	Oxic or repeatedly re-aerated soils; carbon limitation; sulfide reoxidation; unstable reduced zones	Metal sulfide precipitation and alkalinity generation vs. weak remediation efficiency or transient sulfide formation	Ayangbenro et al. (2018), Yang et al. (2023), Tran et al. (2021), Bagheri Novair et al. (2024), Mafane et al. (2025)
Organic sulfur biotransformation by heterotrophs/fungi	Accessible coal matrix; shorter diffusion paths; good microbe-substrate contact; controlled moisture and residence time	Dense aromatic matrices; buried sulfur sites; heterogeneous unmanaged wastes; limited contact and transport	Selective organic sulfur removal vs. low treatment efficiency	Ai et al. (2026), Martínez et al. (2022), Mishra et al. (2016), Nussipov et al. (2026)

## 6 Coal gangue as an interface between biodesulfurization and soil remediation

Coal gangue is a practical interface between coal biodesulfurization and soil science because it is both a sulfur-bearing waste material and a potential reclamation substrate. Generated during coal mining and washing, it commonly contains residual carbonaceous material, clay minerals, pyrite, sulfate minerals, and trace metals, and is often stockpiled (Sun Y. et al., 2025; Qin et al., 2023). Its environmental behavior is shaped by sulfur content, mineral composition, particle size, weathering state, pH buffering capacity, and microbial activity (Yu et al., 2025; Jia et al., 2025).

When appropriately characterized and conditioned, coal gangue can support soil remediation and reclamation by improving porosity, nutrient status, and soil structure. However, it also poses risks of secondary pollution through acid generation and heavy metal leaching. Primary studies illustrate both outcomes: excess sulfur and Fe from abandoned gangue can degrade soil and vegetation (Ma et al., 2020), high-sulfur gangue can release heavy metals during weathering (Dong et al., 2023), whereas bacterial-treated gangue can improve alfalfa germination and growth (Wang et al., 2024). These findings reinforce the need to address harmful constituents and leaching risks before environmental application.

High-sulfur coal gangue is of particular interest. In alkaline or soda-saline soils, controlled sulfur oxidation from high-sulfur gangue can lower pH and improve sodicity-related indicators (Bao et al., 2016). Li et al. and Wang et al. report that, under controlled conditions and after contaminant-risk assessment, amendments with high-sulfur coal gangue reduced saline-alkali soil pH and exchangeable sodium percentage (Li S. et al., 2024; Wang J. et al., 2023).

Coal gangue-based amendments can likewise yield measurable gains in soil and plant performance. In sandy soil, bacterial-treated gangue increased alfalfa germination from 70.0% ± 3.33% to 81.7% ± 5.16%, root length from 35.56 ± 3.18 to 46.4 ± 4.22 mm, and plant height from 42.5 ± 2.13 to 60.3 ± 3.66 mm (Wang et al., 2024). In saline-alkali soil, gangue cover increased saturated water content by 11.1%–17.23%, raised organic matter by 4.99–13.64 mg kg^−1^ and total nitrogen by 0.07–0.12 mg kg^−1^, and improved ryegrass height by up to 16.24 cm (Li S. et al., 2024).

These findings point to a practical translational strategy: biodesulfurization-inspired treatments may improve the safety of coal gangue before soil application, while soil science can help determine when sulfur oxidation is beneficial and when it creates unacceptable risks.

A practical framework could include biological pre-treatment of high-sulfur gangue to reduce pyrite content, followed by washing or neutralization, leachability assessment, blending with organic amendments or alkaline materials, and subsequent evaluation of plant growth and microbial recovery in greenhouse and field trials. This framework should ultimately be translated into risk-based reuse criteria for coal mine solid waste, including thresholds for sulfur loading, acid-generation potential, metal leachability, amendment demand, and post-application monitoring.

## 7 Critical methodological issues: sulfur mass balance and field relevance

A significant limitation in both coal biodesulfurization and mine-soil sulfur research is incomplete sulfur accounting. Although many studies report total sulfur removal, they often do not specify where sulfur goes after treatment. It may be converted into dissolved sulfate, retained on mineral surfaces, incorporated into microbial biomass, precipitated as secondary minerals, emitted as volatile sulfur compounds, or redistributed among solid fractions. Future studies should therefore report a more complete sulfur balance, including the following components.

1. Initial total sulfur in coal or soil;
2. Quantification of pyritic, sulfate, and organic sulfur fractions;
3. Sulfur remaining in the solid phase after treatment;
4. Concentrations of sulfate and other sulfur species present in solution;
5. Measurements of pH, redox potential, Fe^2+^/Fe^3+^ ratios, and concentrations of dissolved metals;
6. Sulfur content in microbial biomass, where quantification is feasible;
7. Plant sulfur uptake, specifically in soil-based studies.

Multi-omics and community-level biological analyses have proven especially valuable in soil studies (Table 3). For example, a high-sulfur coal mining afforestation study combined microbial community analysis with metabolomics to elucidate sulfur-driven biochemical recovery (Wang Y.-W. et al., 2023). Similar integrative approaches should be adopted more widely in coal biodesulfurization and soil remediation research to link sulfur speciation, microbial function, plant response, and contaminant stabilization.

**TABLE 3:** Recommended characterization methods.

Method	Use in coal biodesulfurization	Use in mine-soil remediation	Ref.
Total sulfur analysis	Measures overall sulfur removal	Tracks sulfur accumulation or loss	Huggins (2002), Wang Q. et al. (2022), ISO 334 (2020)
Sulfur speciation	Separates pyritic, sulfate, organic sulfur	Identifies acid-generating risk	Huggins, 2002; ISO 157 (2025)
XPS	Surface sulfur chemical states	Surface oxidation and metal binding	Ai et al. (2026), Kumar et al. (2025)
FTIR	Coal functional groups	Organic matter and mineral changes	Zhao et al. (2023), Ye et al. (2018)
XRD	Pyrite and secondary minerals	Mineral weathering and sulfate minerals	Dong et al. (2024), Wang et al. (2024), Hong et al. (2013)
SEM-EDS	Surface erosion and elemental mapping	Particle weathering and coatings	Dong et al. (2024), Mol et al. (2020)
Ion chromatography	Sulfate release	Sulfate leaching and salinity	Yang et al. (2023), Lewińska-Preis et al. (2021)
ICP-OES/ICP-MS	Fe and trace metal release	Heavy-metal risk assessment	Gani et al. (2025)
16S/metagenomics	Microbial sulfur pathways	Community succession and functional genes	Wang Y.-W. et al. (2023), Hu et al. (2025), Wang Y. et al. (2022)
Metabolomics	Limited use in coal studies	Sulfur metabolites and rhizosphere processes	Wang Y.-W. et al. (2023), Hirschler et al. (2021)
Plant assays	Rare in coal studies	Essential for reclamation evaluation	Yang et al. (2023), Wang et al. (2024)

To complement the qualitative synthesis above, representative quantitative outcomes from selected studies are summarized in Table 4. These values are not directly comparable across systems because coal rank, sulfur speciation, treatment duration, and analytical endpoints differ, but they help define the scale of reported effects.

**TABLE 4:** Representative quantitative outcomes reported in coal biodesulfurization and sulfur management studies.

Study focus	Representative quantitative outcome	Ref.
Pyritic sulfur biooxidation	Under 30 °C, 120 mesh, pH 2.0, and 15 mL inoculum, microbial desulfurization reached 75.12%; coal ignition point increased by 50 °C and activation energy rose from 8,934.3 to 31,464.4 J mol^−1^	Zhao et al. (2023)
Coal matrix effect on organic sulfur removal	Organic sulfur removal reached 60.9% in one coal but only 17.6% in another, a 43.3 percentage-point gap and ∼3.5-fold difference under the same microbial treatment	Ai et al. (2026)
SRB-assisted mine-soil remediation	SRB treatment reduced sulfate values by 52.1%, lowered ORP from 226.85 ± 14.64 to 67.42 ± 4.04 mV, increased pH from 6.25 ± 0.53 to 7.12 ± 0.28, and raised residual metal fractions from 23.47% to 75.98%	Yang et al. (2023)
Sulfur-oxidizing bacteria and plant nutrition	The best-performing isolate produced 311.43 mg L^−1^ sulfate after 21 days; pigeonpea shoot length increased from 43.25 to 59.92 cm and shoot dry weight from 3.50 to 6.95 g after 60 days	Malviya et al. (2022)
Bacterial-treated coal gangue and alfalfa	Seed germination increased from 70.0% ± 3.33% to 81.7% ± 5.16%; root length rose from 35.56 ± 3.18 to 46.4 ± 4.22 mm, and plant height from 42.5 ± 2.13 to 60.3 ± 3.66 mm	Wang et al. (2024)
Coal gangue cover in saline-alkali soil	Saturated water content increased by 11.1%–17.23%, organic matter by 4.99–13.64 mg kg^−1^, total nitrogen by 0.07–0.12 mg kg^−1^, and ryegrass height by up to 16.24 cm	Li S. et al. (2024a)

Table 4 is intended as a benchmark synthesis rather than a direct ranking of performance, because the underlying studies differ in coal rank, sulfur form, assay design, and reporting basis.

## 8 Main research gaps

1. *Coal biodesulfurization research remains insufficiently connected to mine-soil outcomes.* Most coal biodesulfurization studies focus on sulfur removal efficiency. Few studies evaluate whether treated coal waste, process leachates, or introduced microbial systems improve or impair soil restoration outcomes. A soil-science perspective would require testing pH, electrical conductivity, metal leaching, microbial diversity, enzyme activity, plant germination, and long-term sulfur cycling.
2. *DBT model systems only partially represent real coal or soil environments.* The DBT 4 S pathway provides insight into organic sulfur biotransformation; however, real coal contains diverse sulfur forms within a complex macromolecular matrix. Recent findings demonstrating significant variation in *Pseudomonas putida*-mediated organic sulfur removal across structurally distinct coals underscore the need for matrix-aware biodesulfurization approaches (Ai et al., 2026).
3. *Beneficial and harmful effects of sulfur oxidation are not sufficiently distinguished.* Controlled sulfur oxidation can benefit alkaline or soda-saline soils by reducing pH and enhancing sulfate availability, yet it may adversely affect neutral or acidic mine soils by inducing acidification and mobilizing metals. Future research should establish safe operational parameters for sulfur oxidation, considering soil buffering capacity, sulfur loading, redox conditions, and plant tolerance. These operational windows should explicitly incorporate oxygen exposure, water-table position, hydrologic connectivity, pH buffering capacity, sulfur loading, and the stability of reduced products during later re-oxidation events.
4. *Sulfate reduction remains insufficiently integrated into coal biodesulfurization frameworks.* Coal biodesulfurization research predominantly focuses on oxidation processes, whereas mine-soil remediation frequently necessitates reduction, particularly in sulfate-rich and metal-contaminated environments. SRB-based strategies should be integrated with coal sulfur research, as sulfate and metals are prevalent products of pyrite oxidation (Yang et al., 2023).
5. *Field-scale validation of biodesulfurization-inspired approaches remains limited.* Most microbial coal desulfurization studies are conducted at the laboratory scale. While some soil remediation research includes pot experiments or field observations, controlled long-term field trials are scarce.

## 9 Future research directions

1. *Future studies should compare untreated gangue, biologically desulfurized gangue, chemically neutralized gangue, and organically amended gangue within the same soil system.* Such comparisons would clarify whether microbial pretreatment enhances soil safety and fertility.
2. *Coal biodesulfurization studies should incorporate soil-relevant endpoints.* Measuring only total sulfur removal is insufficient; researchers should also assess leachate sulfate, pH, iron, toxic metals, and plant response.
3. *Soil remediation studies should apply sulfur speciation with greater precision.* Many investigations measure total sulfur without distinguishing among pyritic, sulfate, and organic sulfur. Without such differentiation, predicting acid generation or plant sulfur availability remains challenging.
4. *Omics tools should be combined with geochemical and plant-performance measurements to connect microbial identity with function.* Metagenomics, metatranscriptomics, proteomics, and metabolomics can reveal whether sulfur oxidation, sulfate reduction, organic sulfur metabolism, and metal resistance pathways are active in coal-impacted soils. However, these approaches are most informative when linked to sulfur mass balances, redox measurements, contaminant mobility, and plant outcomes.
5. *Field trials should evaluate microbial sulfur management under realistic reclamation conditions.* Factors such as seasonal wetting and drying, oxygen gradients, plant root development, and long-term weathering may substantially alter sulfur cycling relative to laboratory systems.
6. *Field and greenhouse studies should define practical risk-mitigation criteria for re-using coal mine solid waste,* including acceptable sulfur loading, acid-generation potential, metal leachability, amendment requirements, and long-term monitoring triggers.

## 10 Conclusion

Coal biodesulfurization and mine-soil remediation are typically treated as distinct research areas, yet both are linked by microbial sulfur transformation. During coal treatment, microbial oxidation and organic sulfur biotransformation reduce sulfur content before combustion. In coal-impacted soils, analogous processes regulate acid generation, sulfate accumulation, metal mobility, plant sulfur nutrition, and the success of reclamation efforts.

The primary challenge is not simply to maximize microbial sulfur transformation but to control its direction, intensity, and environmental consequences. Pyrite oxidation is beneficial in reactor settings but detrimental in mine spoils. Sulfur oxidation can improve some alkaline or soda-saline soils, yet it may acidify weakly buffered soils. Sulfate reduction immobilizes metals, but it depends on suitable anaerobic conditions and sufficient carbon. Organic sulfur transformation may work well with model compounds, but its performance in real coal is often constrained by sulfur accessibility, matrix structure, and enzyme-substrate compatibility.

In operational terms, beneficial outcomes depend on controlled oxygen exposure, adequate alkalinity or buffering, hydrologic containment of sulfate-rich leachates, and, for sulfate-reducing systems, persistent anoxia with sufficient labile carbon and minimal risk of later re-oxidation.

The quantitative range compiled in this review, from 17.6% to 60.9% organic sulfur removal across different coal matrices, up to 75.12% sulfur removal under optimized microbial treatment, and a rise in residual metal fractions from 23.47% to 75.98% in SRB-amended soils–underscores how strongly performance depends on matrix structure and environmental context (Yang et al., 2023; Ai et al., 2026; Zhao et al., 2023).

Consequently, the future direction of this field should extend beyond the concept of “microbial coal desulfurization.” A more innovative and impactful approach is controlled microbial sulfur management at the interface between coal waste, soil, water, plants, and microbial communities. This strategy integrates coal chemistry, microbial biotechnology, soil science, mine reclamation, and environmental risk assessment. By shifting the focus from fuel-centered biodesulfurization to soil-aware sulfur management, this framework positions coal biodesulfurization as part of a broader reclamation and risk-control strategy for sulfur-rich coal wastes. This is the principal advance of the present review over earlier coal biodesulfurization reviews: it treats microbial sulfur transformation not only as a coal-cleaning mechanism, but also as a coupled driver of waste conditioning, soil chemistry, contaminant behavior, and reclamation success.

## References

[B1] Abdul Rahman N. S. N. Abdul Hamid N. W. Nadarajah K. (2021). Effects of abiotic stress on soil microbiome. Int. J. Mol. Sci. 22, 9036. 10.3390/ijms22169036 34445742 PMC8396473

[B2] Ai C. Cui J. Sun P. Liu C. (2026). Study on the effects and mechanisms of different coal matrix structures on the desulfurization efficiency of Pseudomonas putida. Front. Microbiol. 17. 10.3389/fmicb.2026.1820915 PMC13125091 42063511

[B3] Akimbekov N. S. Digel I. Tastambek K. T. Marat A. K. Turaliyeva M. A. Kaiyrmanova G. K. (2022). Biotechnology of microorganisms from coal environments: from environmental remediation to energy production. Biol. (Basel) 11, 1306. 10.3390/biology11091306 36138784 PMC9495453

[B4] Ayangbenro A. S. Olanrewaju O. S. Babalola O. O. (2018). Sulfate-reducing bacteria as an effective tool for sustainable acid mine bioremediation. Front. Microbiol. 9, 1986. 10.3389/fmicb.2018.01986 30186280 PMC6113391

[B5] Bagheri Novair S. Biglari Z. Asgari Lajayer B. Shu W. Price G. W. (2024). The role of sulphate-reducing bacteria (SRB) in bioremediation of sulphate-rich wastewater: focus on the source of electron donors. Process Saf. Environ. Prot. 184, 190–207. 10.1016/j.psep.2024.01.103

[B6] Bao S. Wang Q. Bao X. Li M. Wang Z. (2016). Biological treatment of saline-alkali soil by Sulfur-oxidizing bacteria. Bioengineered 7, 372–375. 10.1080/21655979.2016.1226664 27558517 PMC5060977

[B7] Chen Z. Xun L. Xia Y. Gong X. (2025). Overlooked role of heterotrophic prokaryotes in sulfur oxidation makes the sediment of the bohai sea a sufficient sink of hydrogen sulfide. mBio 16, e0172225. 10.1128/mbio.01722-25 40622149 PMC12345210

[B8] Calkins W. H. (1994). The chemical forms of sulfur in coal: a review. Fuel 73, 475–484. 10.1016/0016-2361(94)90028-0

[B9] Çelik P. A. Aksoy D. Ö. Koca S. Koca H. Çabuk A. (2019). The approach of biodesulfurization for clean coal technologies: a review. Int. J. Environ. Sci. Technol. 16, 2115–2132. 10.1007/s13762-019-02232-7

[B10] Das P. Roy K. Barbora L. Moholkar V. S. (2024). Interactive effects in biodesulfurization of model oil containing dibenzothiophene and 4,6-dimethyl dibenzothiophene using Rhodococcus rhodochrous MTCC 3552. Bioresour. Technol. Rep. 26, 101842. 10.1016/j.biteb.2024.101842

[B11] Dinh T. Kovacs H. Dobo Z. (2025). A comprehensive review on desulfurization of coal. Energy Convers. Manag. X 27, 101201. 10.1016/j.ecmx.2025.101201

[B12] Dong Y. Lu H. Lin H. (2023). Release characteristics of heavy metals in high-sulfur coal gangue: influencing factors and kinetic behavior. Environ. Res. 217, 114871. 10.1016/j.envres.2022.114871 36423666

[B13] Dong Y. Gao Z. Di J. Wang D. Yang Z. Guo X. (2024). Study on the effectiveness of sulfate-reducing bacteria to remove Pb(II) and Zn(II) in tailings and acid mine drainage. Front. Microbiol. 15 10.3389/fmicb.2024.1352430 38618484 PMC11010684

[B14] Dong J. Zhang W. Wang P. Yi P. Huang Y. Wang Y. (2026). Leveraging environmental applications and risks of coal gangue: a critical review on authigenic inorganic heavy metals, organic contaminants, and the removal of exogenetic contaminants. J. Environ. Sci. 168, 279–291. 10.1016/j.jes.2026.04.012 42680385

[B15] Ghassa S. Noaparast M. Shafaei Sied Z. Abdollahi H. Gharib F. Magdouli S. (2022). Optimization of pyrite bio-oxidation to produce ferric reagent for sphalerite leaching. J. Hazard. Toxic, Radioact. Waste 26, 04021035. 10.1061/(ASCE)HZ.2153-5515.0000644

[B16] Gani A. Desvita H. Mahidin B. Mamat R. Rosdi S. M. (2025). Investigation of heavy metal concentrations for biocoke by using ICP-OES. Results Eng. 25, 103717. 10.1016/j.rineng.2024.103717

[B17] Gao L. Liu Y. Xu K. Bai L. Guo N. Li S. (2024). A short review of the sustainable utilization of coal gangue in environmental applications. RSC Adv. 14, 39285–39296. 10.1039/D4RA06071G 39670158 PMC11635601

[B18] Garbacz A. Nowak A. Marzec-Grządziel A. Przybyś M. Gałązka A. Jaroszuk-Ściseł J. (2025). Impact of coal waste rock on biological and physicochemical properties of soils with different agricultural uses. Sustainability 17, 2603. 10.3390/su17062603

[B19] He H. Hong F.-F. Tao X.-X. Li L. Ma C.-Y. Zhao Y.-D. (2012). Biodesulfurization of coal with Acidithiobacillus caldus and analysis of the interfacial interaction between cells and pyrite. Fuel Process. Technol. 101, 73–77. 10.1016/j.fuproc.2012.04.006

[B20] Hirschler A. Carapito C. Maurer L. Zumsteg J. Villette C. Heintz D. (2021). Biodesulfurization induces reprogramming of sulfur metabolism in Rhodococcus qingshengii IGTS8: proteomics and untargeted metabolomics. Microbiol. Spectr. 9, e0069221. 10.1128/Spectrum.00692-21 34468196 PMC8557817

[B21] Hong F. F. He H. Liu J. Y. Tao X. X. Zheng L. Zhao Y. D. (2013). Comparison analysis of coal biodesulfurization and coal's pyrite bioleaching with Acidithiobacillus ferrooxidans. ScientificWorldJournal 2013, 184964. 10.1155/2013/184964 24288464 PMC3826568

[B22] Hu H. Li S. Wang C. (2025). Sulfur cycle-associated microbial community and functional gene network modulation by vermicompost amendment of saline-alkali soils. Pedosphere. 35 (4), 658–670. 10.1016/j.pedsph.2025.04.005

[B23] Huggins F. E. (2002). Overview of analytical methods for inorganic constituents in coal. Int. J. Coal Geol. 50, 169–214. 10.1016/S0166-5162(02)00118-0

[B24] ISO 157 (2025). Coal — Determination of Forms of Sulfur.

[B25] ISO 334 (2020). Coal and Coke — Determination of Total Sulfur — Eschka Method.

[B26] Jia Y. Lan M. Peng S. Zhang W. Si C. Yu J. (2025). Sustainable utilization of coal gangue in asphalt pavement: a review on design, mechanism, and performance. Mater. (Basel) 18, 5666. 10.3390/ma18245666 PMC12734875 41470438

[B27] Jiao Y. Zhang C. Su P. Tang Y. Huang Z. Ma T. (2023). A review of acid mine drainage: formation mechanism, treatment technology, typical engineering cases and resource utilization. Process Saf. Environ. Prot. 170, 1240–1260. 10.1016/j.psep.2022.12.083

[B28] Kotelnikov V. I. Saryglar C. A. Chysyma R. B. (2020). Microorganisms in coal desulfurization. Appl. Biochem. Microbiol. 56, 521–525. 10.1134/S0003683820050105

[B29] Koyunoğlu C. Karaca H. (2023). Microbial desulphurisation of coal: a review. Int. J. Sustain. Energy 42, 1–24. 10.1080/14786451.2023.2167998

[B30] Koźmińska A. Hassan M. A. Halecki W. Kruszyna C. Hanus-Fajerska E. (2024). Beneficial microorganisms: sulfur-oxidizing bacteria modulate salt and drought stress responses in the halophyte Plantago coronopus L. Sustainability 16, 10866. 10.3390/su162410866

[B31] Kumar S. Chakraborty S. Ghosh S. Banerjee S. Mondal G. Roy P. K. (2024). Revealing soil microbial ecophysiological indicators in acidic environments laden with heavy metals via predictive modeling: understanding the impacts of Black diamond excavation. Sci. Total Environ. 923, 171454. 10.1016/j.scitotenv.2024.171454 38438038

[B32] Kumar V. Verma S. K. Sahu G. Tiwari P. (2025). Ultrasonication-assisted coal desulfurization: mechanisms, process optimization, and industrial applications for sustainable energy. Next Res. 2, 101009. 10.1016/j.nexres.2025.101009

[B33] Lewińska-Preis L. Szram E. Fabiańska M. J. Nádudvari Á. Misz-Kennan M. Abramowicz A. (2021). Selected ions and major and trace elements as contaminants in coal-waste dump water from the lower and upper Silesian coal basins (poland). Int. J. Coal Sci. and Technol. 8, 790–814. 10.1007/s40789-021-00421-9

[B34] Li W. Feng Q. Southam G. Jin T. Li Z. (2023). Changes in microbial community structure during the biooxidation of iron and inorganic/organic sulfur provide prediction of acid mine drainage from coal spoil. Sci. Total Environ. 894, 164945. 10.1016/j.scitotenv.2023.164945 37336403

[B35] Li S. Li X. Qiang X. Yu Z. Li H. Sun Z. (2024). Improving saline–alkaline soil and ryegrass growth with coal gangue treatments. Plants 13, 3419. 10.3390/plants13233419 39683212 PMC11644757

[B36] Li Y. Cao Y. Ruan M. Li R. Bian Q. Hu Z. (2024a). Mechanism and *in situ* prevention of oxidation in coal gangue piles: a review aiming to reduce acid pollution. Sustainability 16, 7208. 10.3390/su16167208

[B37] Li Y. Liang Z. Yan X. Qin T. Wu Z. Zheng C. (2024b). The sulfur conversion functional microbial communities in biogas liquid can participate in coal degradation. Pol. J. Microbiol. 73, 315–327. 10.33073/pjm-2024-027 39214142 PMC11398273

[B38] Ma J. Quan Z. Sun Y. Du J. Liu B. (2020). Excess sulfur and Fe elements drive changes in soil and vegetation at abandoned coal gangues, Guizhou China. Sci. Rep. 10, 10456. 10.1038/s41598-020-67311-z 32591606 PMC7320150

[B39] Mafane D. Ngulube T. Mphahlele-Makgwane M. M. (2025). Anaerobic bioremediation of acid mine drainage using sulphate-reducing bacteria: current status, challenges, and future directions. Sustainability 17, 3567. 10.3390/su17083567

[B40] Malviya D. Varma A. Singh U. B. Singh S. Singh H. V. Saxena A. K. (2022). Sulfur-oxidizing bacteria from coal mine enhance sulfur nutrition in pigeonpea (Cajanus cajan L.). Front. Environ. Sci. 10 10.3389/fenvs.2022.932402

[B41] Martínez I. Mohamed M.E.-S. García J. L. Díaz E. (2022). Enhancing biodesulfurization by engineering a synthetic dibenzothiophene mineralization pathway. Front. Microbiol. 13 10.3389/fmicb.2022.987084 36274708 PMC9579287

[B42] Mishra S. Pradhan N. Panda S. Akcil A. (2016). Biodegradation of dibenzothiophene and its application in the production of clean coal. Fuel Process. Technol. 152, 325–342. 10.1016/j.fuproc.2016.06.025

[B43] Mol A. R. van der Weijden R. D. Klok J. B. M. Buisman C. J. N. (2020). Properties of sulfur particles formed in biodesulfurization of biogas. Minerals 10, 433. 10.3390/min10050433

[B44] Monterroso C. Macías F. (1998). Drainage waters affected by pyrite oxidation in a coal mine in Galicia (NW Spain): composition and mineral stability. Sci. Total Environ. 216, 121–132. 10.1016/S0048-9697(98)00149-1

[B45] Moyo A. Amaral Filho J. R. d. Harrison T. L. Broadhurst J. L. (2019). Implications of sulfur speciation on the assessment of acid rock drainage generating potential: a study of South African coal processing wastes. Minerals 9, 776. 10.3390/min9120776

[B46] Nancucheo I. Johnson D. B. (2011). Significance of microbial communities and interactions in safeguarding reactive mine tailings by ecological engineering. Appl. Environ. Microbiol. 77, 8201–8208. 10.1128/aem.06155-11 21965397 PMC3233063

[B47] Nguyen P. M. Do P. T. Pham Y. B. Doan T. O. Nguyen X. C. Lee W. K. (2022). Roles, mechanism of action, and potential applications of sulfur-oxidizing bacteria for environmental bioremediation. Sci. Total Environ. 852, 158203. 10.1016/j.scitotenv.2022.158203 36044953

[B48] Ningombam L. Mana T. Pradhan S. Apum G. Singh Y. D. (2025). Fungal bioremediation in environmental pollution and recent strategies. Discov. Environ. 3, 100. 10.1007/s44274-025-00267-x

[B49] Novak J. M. Ippolito J. A. Ducey T. F. Watts D. W. Spokas K. A. Trippe K. M. (2018). Remediation of an acidic mine spoil: miscanthus biochar and lime amendment affects metal availability, plant growth, and soil enzyme activity. Chemosphere 205, 709–718. 10.1016/j.chemosphere.2018.04.107 29729625 PMC7904245

[B50] Nussipov D. Akimbekov N. Tastambek K. Digel I. Aimagambetov A. Xiangrong L. (2026). Resource recovery from low-rank coal and livestock manure for sustainable agroecosystems: a review. Arch. Microbiol. 208, 204. 10.1007/s00203-026-04758-0 41711903 PMC12920336

[B51] Pan Y. Tan T. Ren R. Meng J. Yu N. Jin X. (2026). Soil organic carbon decomposition in response to moisture, microbial communities, and biochar addition in alfisols. Biochar 8, 5. 10.1007/s42773-025-00513-8

[B52] Patsis A. C. Schuler C. J. Toner B. M. Santelli C. M. Sheik C. S. (2025). The potential for coupled organic and inorganic sulfur cycles across the terrestrial deep subsurface biosphere. Nat. Commun. 16, 3827. 10.1038/s41467-025-59241-z 40268922 PMC12019592

[B53] Prayuenyong P. (2002). Coal biodesulfurization processes. Songklanakarin J. Sci. Technol. 24, 494.

[B54] Qin Q. Geng H. Deng J. Su X. Chen M. Park C. B. (2023). Al and other critical metals co-extraction from coal gangue through delamination pretreatment and recycling strategies. Chem. Eng. J. 477, 147036. 10.1016/j.cej.2023.147036

[B55] Sekhohola-Dlamini L. M. Khan S. Wang B. Yu Z. Cowan A. K. (2025). Recent progress on the biological degradation and solubilization of coal. Biodegradation 36, 84. 10.1007/s10532-025-10175-9 40952519 PMC12436530

[B56] Singh V. K. Singh R. Kumar A. Bhadouria R. Notarte K. I. (2021). “Chapter 14 - perspectives in desulfurization of coal using microbes,” in Microbe Mediated Remediation of Environmental Contaminants. Editors Kumar A. Singh V. K. Singh P. Mishra V. K. (Cambridge, United Kingdom: Woodhead Publishing), 141–155.

[B57] Sun Y. Bai B. Yang X. Zhu S. Tian J. Wang Z. (2025). Coal gangue utilization: applications, challenges, and sustainable development strategies. Energies 18, 444. 10.3390/en18020444

[B58] Sun J. Yang J. Wang X. Lu H. Zhong T. Xu H. (2025). Afforestation enhances soil ecosystem multifunctionality by improving soil quality and enzyme activities in coastal saline-alkali land. Biol. (Basel) 14, 1588. 10.3390/biology14111588 41300377 PMC12650132

[B59] Tran T. T. Kannoorpatti K. Padovan A. Thennadil S. (2021). Sulphate-reducing bacteria’s response to extreme pH environments and the effect of their activities on microbial corrosion. Appl. Sci. 11, 2201. 10.3390/app11052201

[B60] Wang R. Lin J. Q. Liu X. M. Pang X. Zhang C. J. Yang C. L. (2018). Sulfur oxidation in the acidophilic autotrophic acidithiobacillus spp. Front. Microbiol. 9, 3290. 10.3389/fmicb.2018.03290 30687275 PMC6335251

[B61] Wang X. Jiang H. Zheng G. Liang J. Zhou L. (2021). Recovering iron and sulfate in the form of mineral from acid mine drainage by a bacteria-driven cyclic biomineralization system. Chemosphere 262, 127567. 10.1016/j.chemosphere.2020.127567 32755692

[B62] Wang Z. Xu Y. Zhang Z. Zhang Y. (2021). Review: Acid mine drainage (AMD) in abandoned coal mines of Shanxi, China. Water 13, 8. 10.3390/w13010008

[B63] Wang Q. Lin Z. Wu Q. Lin L. Zhang Q. (2022). Determination of total sulfur in coal by ultraviolet fluorescence method based on the large capacity combustion framework. Fuel Commun. 11, 100063. 10.1016/j.jfueco.2022.100063

[B64] Wang Y. Bi H. Y. Chen H. G. Zheng P. F. Zhou Y. L. Li J. T. (2022). Metagenomics reveals dominant unusual sulfur oxidizers inhabiting active hydrothermal chimneys from the southwest Indian ridge. Front. Microbiol. 13, 861795. 10.3389/fmicb.2022.861795 35694283 PMC9174799

[B65] Wang Y.-W. Bai D.-s. Yang X. Zhang Y. Luo X. (2023). Soil sulfur cycle bacteria and metabolites affected by soil depth and afforestation conditions in high-sulfur coal mining areas. Appl. Soil Ecol. 185, 104802. 10.1016/j.apsoil.2022.104802

[B66] Wang J. Zhao A. Ma F. Liu J. Xiao G. Xu X. (2023). Amendment of saline–alkaline soil with flue-gas desulfurization gypsum in the yinchuan plain, northwest China. Sustainability 15, 8658. 10.3390/su15118658

[B67] Wang Y. Liu M. Di Z. Cao W. He S. (2024). Feasibility analysis of bacterial-treated coal gangue for soil improvement: growth-promoting effects of alfalfa. Minerals 14, 676. 10.3390/min14070676

[B68] Xie P. Xu X.-J. Zhang Q. Hou Y.-Y. Fan K.-L. Zhang R.-C. (2025). Potent and selective inhibition of sulfate-reducing bacteria by neutral red. Environ. Sci. and Technol. 59, 6115–6125. 10.1021/acs.est.4c09915 39972257

[B69] Xu J. Liu X. Wu J. Zhang Y. (2022). An effective method to remove organic sulfur in coal: effects on the physicochemical properties and combustion kinetics. Environ. Prog. and Sustain. Energy 41, e13779. 10.1002/ep.13779

[B70] Yang Z. Wu Q. Liu Z. Qi X. Zhang Z. He M. (2023). Harnessing sulfate-reducing bacteria with plants growing to revitalize metal-tainted coal mine soils in midwest China: metal sequestration performance, ecological networking interaction, and functional enzymatic prediction. Front. Microbiol. 14 10.3389/fmicb.2023.1306573 38033581 PMC10684746

[B71] Ye J. Zhang P. Zhang G. Wang S. Nabi M. Zhang Q. (2018). Biodesulfurization of high sulfur fat coal with Indigenous and exotic microorganisms. J. Clean. Prod. 197, 562–570. 10.1016/j.jclepro.2018.06.223

[B72] Yi Q. Wu S. Southam G. Robertson L. You F. Liu Y. (2021). Acidophilic Iron- and sulfur-oxidizing bacteria, Acidithiobacillus ferrooxidans, drives alkaline pH neutralization and mineral weathering in Fe ore tailings. Environ. Sci. and Technol. 55, 8020–8034. 10.1021/acs.est.1c00848 34043324

[B73] Yu S. Li J. Du D. Li H. Hao J. Teng Z. (2025). Coal gangue ecological matrix coupled with microalgae for soil improvement and plant growth in reclaimed mining areas. Biol. (Basel) 14, 741. 10.3390/biology14070741 40723302 PMC12292427

[B74] Yuan D. Wei Y. Fan X. Liu F. (2025). Difference in biological oxidation between high-sulfur coal and pure pyrite at different pH levels. Separations 12, 66. 10.3390/separations12030066

[B75] Zhang S. Wu P. Zhang J. Li Q. Luo C. (2025). Effects of acid mine drainage on microbial community development and physicochemical properties of mine contaminated sites in southwest China. Sci. Rep. 15, 20776. 10.1038/s41598-025-05799-z 40594805 PMC12218272

[B76] Zhao D. Sun P.-p. Ai C.-m. Mu X.-z. (2023). Study on the experiment and reaction kinetics of sulfur removal from coal by microorganisms. Front. Microbiol. 14 10.3389/fmicb.2023.1184253 37342566 PMC10277619

